# *Ex Vivo* Cytosolic Delivery of Functional Macromolecules to Immune Cells

**DOI:** 10.1371/journal.pone.0118803

**Published:** 2015-04-13

**Authors:** Armon Sharei, Radiana Trifonova, Siddharth Jhunjhunwala, George C. Hartoularos, Alexandra T. Eyerman, Abigail Lytton-Jean, Mathieu Angin, Siddhartha Sharma, Roberta Poceviciute, Shirley Mao, Megan Heimann, Sophia Liu, Tanya Talkar, Omar F. Khan, Marylyn Addo, Ulrich H. von Andrian, Daniel G. Anderson, Robert Langer, Judy Lieberman, Klavs F. Jensen

**Affiliations:** 1 Department of Chemical Engineering, Massachusetts Institute of Technology, Cambridge, Massachusetts, United States of America; 2 The Ragon Institute of MGH, MIT and Harvard, Cambridge, Massachusetts, United States of America; 3 Department of Microbiology and Immunobiology, Harvard Medical School, Boston, Massachusetts, United States of America; 4 The Koch Institute for Integrative Cancer Research, Massachusetts Institute of Technology, Cambridge, Massachusetts, United States of America; 5 Program in Cellular and Molecular Medicine, Boston Children’s Hospital, Harvard Medical School, Boston, Massachusetts, United States of America; Murdoch University, AUSTRALIA

## Abstract

Intracellular delivery of biomolecules, such as proteins and siRNAs, into primary immune cells, especially resting lymphocytes, is a challenge. Here we describe the design and testing of microfluidic intracellular delivery systems that cause temporary membrane disruption by rapid mechanical deformation of human and mouse immune cells. Dextran, antibody and siRNA delivery performance is measured in multiple immune cell types and the approach’s potential to engineer cell function is demonstrated in HIV infection studies.

## Introduction

Modulating immune cell function through intracellular delivery of biomolecules has many potential applications. Delivery of macromolecules, such as polysaccharides, proteins, or nucleic acids, to the cell cytoplasm can transiently or permanently alter cell function for research or therapeutic purposes. Indeed some promising immunotherapies, such as T cell[1] and dendritic cell[2] adoptive transfer therapies, rely on the manipulation of intracellular processes to generate therapeutic benefit. However, existing techniques for intracellular delivery to primary immune cells, especially resting lymphocytes, have limitations. For example, electroporation results in considerable cellular toxicity, viral vectors are unable to infect resting lymphocytes, and cell membrane penetrating (or transduction) peptides do not efficiently transfect primary lymphocytes [3, 4]. Antibody or aptamer-drug complexes [5–7] and conjugates [8] require specific targeting motifs for each cell type and distinct designs to carry different payloads. Advances in nanoparticle and liposome based technologies have resulted in improved intracellular delivery of drugs and antigens to phagocytic antigen presenting cells, such as dendritic cells and monocyte/macrophages, but are ineffective for other lymphoid cells [9–11]. Indeed most of the listed methods lead to endosomal uptake of their payload [12], and only a small proportion of the target material (estimated as ~1–2%) [13] escapes from the endosome to the cytosol, where it needs to traffic for biological activity. Thus, there is an acute need for alternative techniques capable of efficient and nontoxic delivery of a variety of macromolecules to immune cells.

**Fig 1:**
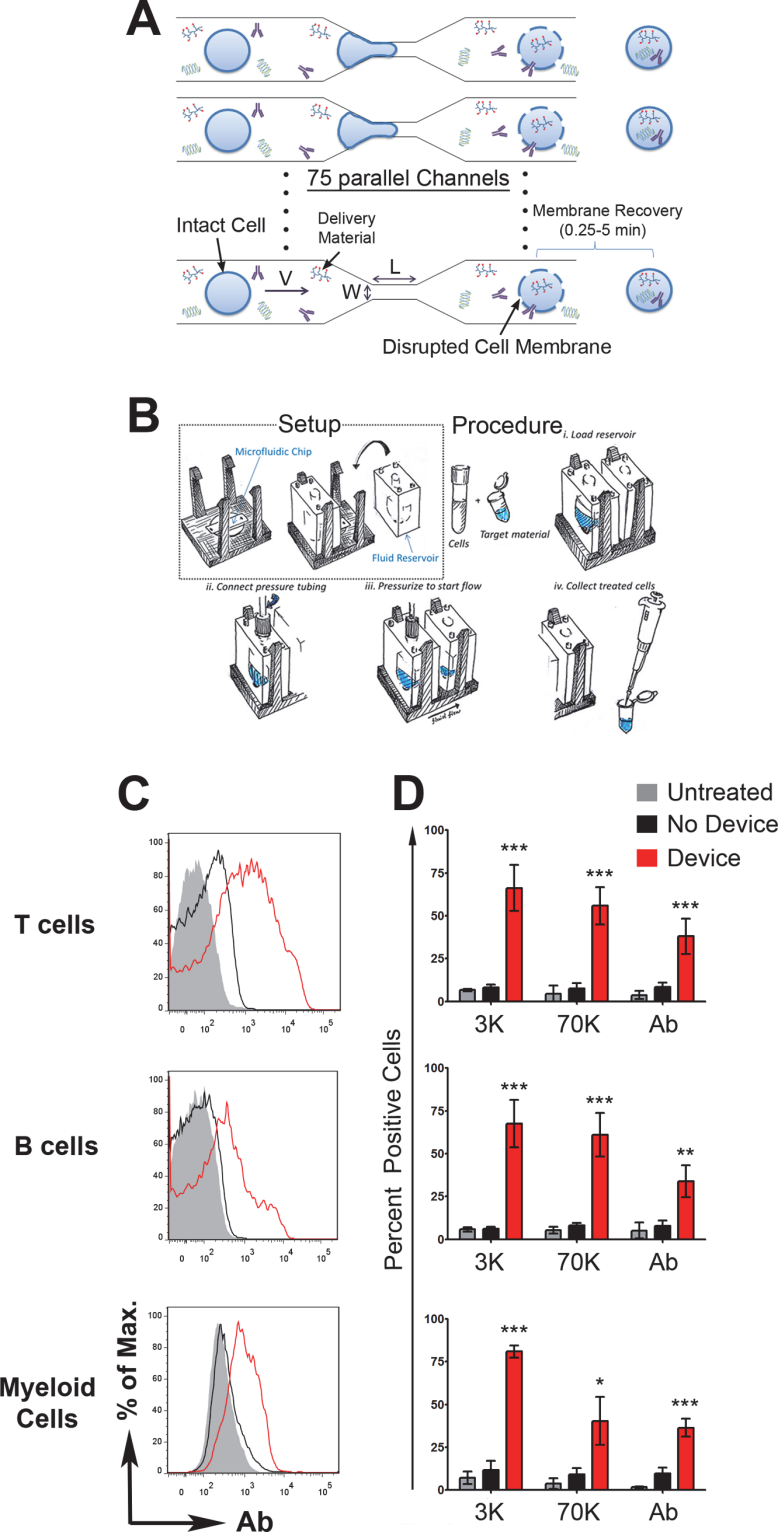
Delivery methodology and performance in mouse cells. **A)** Illustration of device design and delivery mechanism. **B)** Illustration of the system setup and delivery procedure. **C)** Representative histograms of T cells, B cells and myeloid cells (CD11b^+^) treated by the CellSqueeze device to deliver APC-labeled IgG1. **D)** Delivery efficiency of Cascade blue-labeled 3 kDa dextran, fluorescein-labeled 70 kDa dextran, and APC-labeled IgG1. All results were measured by flow cytometry within an hour of treatment. Dead cells were excluded by propidium iodide staining. Viability is shown in **S2 Fig**. Data in **D)** (mean ± SD) are from 3 independent experiments. Untreated cells were not put through the device or exposed to the biomolecules. The ‘no device’ samples were incubated with the biomolecules, but were not treated by the device. This control is meant to account for surface binding, endocytosis and other background effects.

In this work, we sought to adapt a vector-free microfluidic delivery concept, previously demonstrated for use in cell reprogramming and imaging applications[14, 15], to the challenge of intracellular delivery to immune cells. In this delivery system, cells flow from a reservoir into a series of parallel microfluidic channels (**Fig 1A**) and undergo rapid mechanical deformation as they pass through a constriction point in the channel. When the channel constriction is appropriately sized, the deformation transiently disrupts the cell membrane and enables macromolecules present in the surrounding buffer to enter the cell cytosol. Within ~5 min, the membrane recovers its integrity and the macromolecules taken up by the cell remain trapped in the cell cytosol [16].

## Results and Discussion

To modify and implement this approach for immune cells, we fabricated microfluidic devices that consist of 45–75 parallel microfluidic channels of varying constriction lengths (10–50μm), widths (4–9μm) and number of constrictions per channel (1–5 constrictions) (**S1A Table**). The system developed to operate the microfluidic chip consists of a mounting component that secures fluid reservoirs to the silicon and glass device, and a pressure regulation system that controls the gas pressure used to drive the fluid through the system. The operating procedure is illustrated in **Fig 1B**. Our studies were designed to vary constriction length (L), width (W), operating temperature, and fluid speed (V, note that fluid speed is determined by operating pressure) because they had previously been identified as parameters that influence delivery efficiency and cell viability in other cell types(**S1C Table**) [14, 16]. All the buffers we tested (PBS, PBS+2% serum, complete culture media, and whole human blood) were found to be compatible with the system and could flow through the microfluidic channels.

To assess the potential of the fabricated designs to enable intracellular delivery to primary immune cells, mouse T cells, B cells, and monocytes/macrophages were treated by the aforementioned microfluidic chips in the presence of fluorescently labeled dextran (3 and 70 kDa), and antibodies. These materials were selected as models for small molecules, polysaccharides, and proteins. Based on delivery efficiency and viability results, delivery using the 30–4 design (i.e. constriction has a 30 μm length and 4 μm width) was found to be the most effective for lymphocytes and myeloid cells (**Fig 1C and 1D and S1A–S1C Fig**). Simultaneous delivery of dextrans (3 kDa and 70 kDa) and antibody showed that the delivery of these molecules was proportional, i.e. cells that received antibody, also received a comparative amount of dextran molecules (**S1D Fig**). This observation is consistent with the proposed membrane disruption-based delivery mechanism[16].

**Fig 2:**
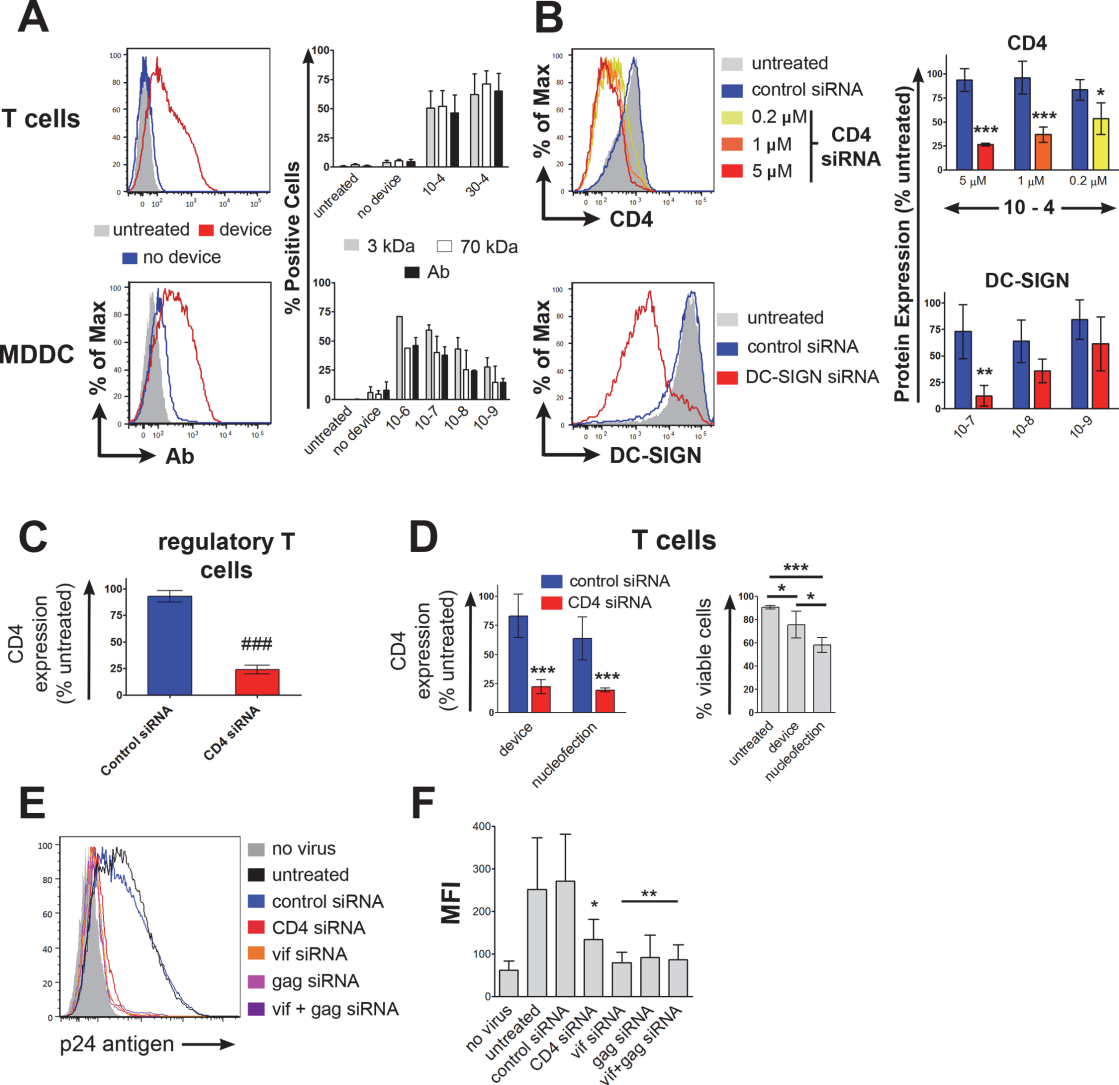
Delivery to human immune cells. **A)** Human T cells and MDDCs were tested for delivery of cascade blue labeled 3kDa dextran, fluorescein labeled 70kDa dextran, and APC labeled IgG1. The representative histograms for a 30–4 (T cells) and 10–7 (MDDCs) device (left) and replicates across device designs (right) are displayed. **B)** SiRNA mediated knockdown of CD4 and DC-SIGN protein levels in CD4^+^ T cells and MDDCs respectively. **C)** Knockdown of CD4 expression in human regulatory T cells in response to treatment by a 30–4 device. Dead cells were excluded for delivery or knockdown analysis. **D)** Comparison of device performance in T cells to nucleofection by Amaxa. Protein expression 72hrs after siRNA delivery and cell viability after treatment are shown. **E)** Intracellular staining for the p24 antigen was used as an indicator of HIV infection level in treated human CD4^+^ T cells 24hrs after infection. In these studies, vif and/or gag, siRNA was delivered 24hrs prior to infection while CD4 siRNA was delivered 48hrs prior to infection. **F)** Median fluorescence intensity of the p24 antigen stain across repeats (min. N = 4) of the experimental conditions. Data are represented as mean + 1 standard error.

The applicability of this approach to human immune cells was verified by testing device designs with constriction widths ranging from 4–6 μm for T cells and 6–9 μm for monocyte-derived dendritic cells (MDDCs). The testing range was determined based on observed differences in delivery behavior during preliminary experiments which indicated that the larger MDDCs required a wider constriction size (**S1B Table**). The most effective designs delivered 3 kDa dextran to 70% ± 9% of T cells (4 μm constriction size) and 60% ± 4.5% of MDDCs (7 μm constriction size) (**Fig 2A and S2A and S2B Fig**). Delivery of fluorescently labeled siRNA (CD45RA siRNA—Alexa-Fluor-488) yielded similar results (**S2C Fig**).

The final delivery protocols for the aforementioned human and murine cell types were developed by varying four key parameters: constriction dimensions, temperature, buffer composition, and pressure (**S1C Table**). Based on our previous work and through the course of these experiments, we noticed the following behavior: i) Narrower, longer constrictions can result in greater delivery efficiency but may negatively impact viability. ii) Treating samples at lower temperatures, e.g. on ice, yields greater delivery efficiency as it likely slows the membrane repair process and provides a longer delivery window[16]. iii) Buffers that lack calcium, e.g. PBS, can facilitate more delivery by preventing the induction of calcium influx-based membrane repair mechanisms. However, prolonged exposure to calcium-free conditions can reduce viability[16]. iv) higher operating pressures corresponded to higher cell speeds in the channels, increased delivery efficiency and lower viability.

To examine this approach’s ability to induce protein knockdown, we delivered siRNA against human CD4 or CD45RA to blood derived T cells and siRNA against DC-SIGN to MDDCs. Flow cytometry and qRT-PCR results at 72hours and 48hours post-delivery respectively showed gene-specific knockdown (**Fig 2B and S2C and S2D Fig**). Device mediated knockdown lasted ~10 days in T cells (**S2D Fig**); consistent with previous findings for gene silencing in T cells [7]. The approach was also found to be applicable to human regulatory T cells (**Fig 2C**), B cells and monocytes (**S3A and S3B Fig**). Comparative experiments with nucleofection (an established electroporation-based delivery technique optimized for nucleic acid delivery to immune cells) demonstrated similar siRNA knockdown levels between the two techniques **(Fig 2D)**, however, cells treated by the microfluidic devices had significantly higher viability post-treatment (P<0.05), 2.5x higher 3kDa dextran delivery, less non-specific knockdown **(S3C Fig)**, and improved long-term viability. Comparison of our platform’s performance to nucleofection in the context of MDDCs yielded similar results to T cells (**S2B Fig**). Moreover, parallel studies conducted by Griesbeck et. al. have shown that protein transcription factors delivered by squeezing are functional and able to induce target gene expression in primary human pDCs. By comparison, there is limited evidence that electroporation can facilitate delivery of functional proteins[17, 18].

Finally, we tested if HIV infection and replication in human primary CD4^+^ T cells can be inhibited by vector-free microfluidic delivery of siRNA targeting viral genes. In experiments with live HIV virus, we observed a significant reduction in infection (p<0.01), as measured by p24 antigen levels, in T cells treated with CD4, vif, or gag[19] targeted siRNA (**Fig 2E and 2F**).

## Conclusion

Despite tremendous progress in drug delivery technology, intracellular delivery of macromolecules to immune cells remains a significant challenge [14, 20, 21]. Results shown here demonstrate the potential of this microfluidic membrane disruption approach to be a robust platform for the delivery of macromolecules to murine and human immune cells. This technology has shown: (i) the ability to deliver a diversity of biologically relevant macromolecules (polysaccharides, proteins, and nucleic acids); (ii) efficacy in most immune cell subsets, including T cells, B cells, DCs, and monocytes/macrophages (Figs **1C and 1D and Fig 2)**; (iii) independence from vector material and electrical fields, thus overcoming some of the challenges associated with endocytic entrapment and electroporation-level toxicity[22]; and (iv) the simultaneous delivery of multiple classes of macromolecules to target cells (**S1D and S2C Figs**). By facilitating effective, vector-free delivery of a diversity of materials, this system could potentially be deployed as a platform for immune cell engineering and enable robust control of cell function for research and clinical applications.

## Materials and Methods

### CellSqueeze microfluidic devices

As described previously [14, 16], the CellSqueeze platform consists of three major components: a) a silicon and glass microfluidic chip that contains multiple channels in parallel, each containing at least one constriction point b) a reservoir system that interfaces with the chip and allows one to load/collect the cell suspension c) a pressure regulation system to pressurize the reservoirs and facilitate fluid flow through the chip. In a typical workflow (**Fig 1A**), one must mix the target delivery material with the desired cells (in suspension) and load them into the reservoir. One must then connect the pressure tubing to the reservoir and pressurize the chamber at the desired level to initiate fluid flow. After treatment, the cells may be collected from the output reservoir and incubated at the desired temperature for 5min to ensure proper membrane recovery before further processing.

CellSqueeze devices and the associated operating equipment were obtained from SQZ Biotechnologies, USA. Devices were assembled and used in accordance with manufacturer protocols and previously described methods [16]. Briefly, individual CellSqueeze devices and the associated reservoir systems were kept in 70% ethanol to maintain sterility. For each experiment, the desired CellSqueeze device was connected to the reservoirs and 70ul of PBS was used to flush the system prior to use with cell samples.

During a delivery experiment, the target cells, device+reservoir, and collection plate are kept on ice (T cells and B cells) or at room temperature (dendritic cells). Cells (at a concentration of 2x10^6^-1x10^7^ cells/ml in PBS or culture media) are mixed with the target delivery material at the desired concentration prior to being added to the fluid reservoir. The pressure tubing is connected, system is set at the desired operating pressure, and the flow is initiated by pressurizing the reservoir containing the sample. After passing through the chip, cells are collected from the collection reservoir and transferred to a 96-well plate. This process is repeated until all experimental conditions are complete. To minimize clogging, the direction of flow in the chip is alternated between samples. Where relevant, samples are allowed to incubate on ice for 5min post-treatment before media is added and they are transferred for further processing.

### Mouse immune cell isolations

All animal work must was conducted according to relevant national and international guidelines. Mouse cells were isolated from 6–8 week male C57BL6/J (Jackson Labs) mice that had been sacrificed by CO_2_ inhalation. These procedures were conducted in accordance with MIT guidelines established by the Committee for Animal Care (CAC) and division of comparative medicine (DCM) under protocol number 1011-125-14 and 0112-005-15. Where live animal injections were performed for macrophage isolation, isoflurane gas was used as an inhaled anesthetic. Animals were monitored regularly by veterinary staff and housed in the central facilities at MIT. All procedures were conducted in accordance with approved procedures in our animal protocol on file with the institutional IACUC. All procedures/studies conducted in this manuscript were approved by our IACUC office (the CAC).

T and B cells were isolated from the spleens of wild-type C57BL6/J mice using cell-specific isolation kits from Stemcell Technologies (Vancouver, Canada) based on manufacturer's instructions (negative selection technique). Monocytes/macrophages were isolated from the peritoneal cavity of wild-type C57BL6/J mice 3 days following intraperitoneal injection of 1ml of thioglycollate solution. Cells were purified using CD11b positive selection kit from Stemcell Technologies (Vancouver, Canada) based on manufacturer's instructions. Cells were cultured in glutamine containing RPMI 1640 media containing 10% fetal bovine serum, 1% antibiotics/antimycotic, 0.5% beta-mercaptoethanol, 1% non-essential amino-acids, 1 mM sodium pyruvate, and 10 mM HEPES buffer (all from (Life Technologies, NY, USA)).

### Human primary T cells and Monocyte Derived Dendritic cells

Human PBMCs were separated using Ficoll-Paque (GE Healthcare, Uppsala, Sweden) density gradient centrifugation from whole blood obtained from Kraft Family Blood Donor Center, Boston, MA according to an Institutional Review Board approved protocol. CD4+ T cells were separated from the CD14-negative fraction of PBMCs using CD14 and CD4 magnetic microbeads (MACS Miltenyi Biotec, Auburn, CA). T cells were cultured in RPMI 1640 media (Cellgro, Manassas, VA) containing 10% Human Serum (AB) (GemCell, West Sacramento, CA), 100 U/ml penicillin and streptomycin sulfate 100 μg/ml (H10 medium) supplemented with 5 ng/ml rhIL-15 (R&D Systems, Minneapolis, MN) to maintain cell viability without cell activation. Human Monocyte derived Dendritic Cells (MDDCs) were prepared from CD14-positive monocytes selected from peripheral blood mononuclear cells using anti-CD14 magnetic microbeads (MACS Miltenyi Biotec) and cultured for 6 days with 100 ng/ml interleukin-4 and 50 ng/ml granulocyte-macrophage colony-stimulating factor (R & D Systems).

### Cell transfection

Human CD45 siRNA: sense 5'-AF488 CUGGCUGAAUUUCAGAGCAdTdT-3', Human CD4 siRNA: sense 5'-GAUCAAGAGACUCCUCAGUdTdT-3' (Alnylam, Cambridge, MA); vif siRNA: sense 5'-CAGAUGGCAGGUGAUGAUUGT-3', gag siRNA: sense 5'-GAUUGUACUGAGAGACAGGCU-3'[19] (GenePharma, Shanghai, China); control scrambled siRNA: 5'-GCCAAGCACCGAAGUAAAUUU-3', Human DC-SIGN siRNA: sense 5'-GGAACUGGCACGACUCCAUUU -3’ (Dharmacon, ThermoScientific, Pittsburgh, PA).

### Nucleofection

In our described electroporation experiments, we used the Amaxa Nucleofector II (Lonza Inc., Allendale, NJ) and followed the manufacturer’s recommendations. Human T cell experiments were conducted using the program for human unstimulated T cells, high viability, U-014 with a human T cell kit. For human MDDCs we used the program for human dendritic cells U-002 with a human dendritic cells kit for MDDCs. Briefly 2x10^6^ cells were suspended in 100 μl of Nucleofection solution with 200 pmol of siRNA and nucleofected by the machine. To test protein delivery, we used an APC-labeled mouse IgG1 (cl. MOPC-21, Biolegend) at 0.02mg/ml for both CellSqueeze and nucleofection experiments. We also used 3 kDa Cascade Blue labeled dextran and 70kD Fluorescein labeled dextran at 0.2 mg/ml (Invitrogen).

### Regulatory T cells

Regulatory T cells (Tregs) were isolated and expanded as previously described [23, 24]. Briefly CD4+ T Cell-enriched PBMC were isolated from peripheral blood of healthy individuals by density centrifugation using the CD4+ T cells RosetteSep enrichment kit (Sigma-Aldrich and STEMCELL Technologies) and labeled with anti-CD3-PE-Cy7, CD4-FITC, CD25-APC and CD127-PE. CD3+CD4+CD25+CD127low Tregs were sorted on a FACS Aria cell sorter (BD Biosciences), stimulated with anti-CD3/anti-CD28-coated microbeads (Invitrogen) and cultured with IL-2 (300 U/ml).

For siRNA delivery, at day 7 of culture, Tregs were washed and resuspended at 1.0 x 10^7^ cells/ml in X-VIVO 15 (Lonza) media alone. 1.0 x 10^6^ cells were used per condition. CD4 siRNA (5’-GAUCAAGAGACUCCUCAGU-3’, Alnylam) and control siRNA (siGENOME Non-Targeting siRNA Pool #1, Thermo Fisher) were used at 1μM with 30–4 chips design at 100 psi.

2 days after siRNA delivery, cells were stained with LIVEDEAD Fixable Violet Dead Cell Stain Kit (Life Technologies) and anti-CD4-APC. Data were acquired on a LSR2 flow cytometer (BD Biosciences) and analyzed on FlowJo (Treestar).

### Flow cytometry

Mouse cells were stained with the following antibodies: anti-CD8-Pacific Blue, anti-CD4-APC, anti-CD11b-PE (cl. M1/70), anti-CD11c-APC. Propidium iodide was used to exclude dead cells. Data was acquired using a FACS CantoII, LSR II, or LSRFortessa (BD Biosciences) and analyzed using FlowJo (Tree Star, Ashland, OR).

Human cells were stained with the following antibodies: anti-CD3-APC (cl.OKT3), anti-CD45RA-PE-Cy7 (cl. HI100) and anti-CD4-AF488 (cl.OKT4) from Biolegend (San Diego, CA) and an anti-DC-SIGN-APC (cl.9E9A8) (R & D Systems, Minneapolis, MN). Dead cells were excluded using Sytox blue and 7-AAD (7-Aminoactinomycin D) dead stain dye (Invitrogen). Data were acquired using a FACS CantoII (BD Biosciences) and analyzed using FlowJo (Tree Star, Ashland, OR).

### HIV infection and intracellular p24 Antigen staining

Primary CD4+ T cells were treated with 5 μM siRNA using a 10–4 chip. For knockdown of CD4, siRNA was delivered 48 hrs prior to infection while siRNA targeting viral genes vif and gag were delivered 24 hrs prior to infection. The cells were then stimulated overnight with 5 μg/ml Phytohaemagglutinin (PHA) and infected with HIVIIIB in 96 well plates at 2×10^5^ cells/well with HIV IIIB (400 ng/ml p24). HIV IIIB was obtained from the NIH AIDS Reagent Program and viral stock was prepared as previously described[25]. The infection was enhanced by the addition of polybrene at 5 μg/ml and spinoculation at 1200 xg, for 2 hrs at 37 °C [26]. Intracellular p24 antigen staining was performed 24 hrs later using an anti-p24 KC57-FITC Antibody (Beckman Coulter, Fullerton, CA) with Fix & Perm Kit for Cell permeabilization (Invitrogen) and analyzed by flow cytometry.

### Quantitative RT-PCR

Total RNA was isolated from T cells using RNeasy Mini Kit (Qiagen) and copy DNA was synthesized using Superscript III and random hexamers (Invitrogen). Real Time PCR was performed using SsoFast EvaGreen Supemix and a Bio-Rad CFX96 Real-Time PCR System (Bio-Rad Laboratories, Hercules, CA). The primers were as follows: Gapdh forward: 5’- AGCCACATCGCTCAGACAC -3’, Gapdh reverse: 5’- GCCCAATACGACCAAATCC -3’, CD4 forward: 5’- GGCAGTGTCTGCTGAGTGAC—3’, CD4 reverse: 5’- GACCATGTGGGCAGAACCT—3’.

### Statistical analysis

One-way analysis of variance (ANOVA) with Bonferroni's Multiple comparison test was performed when comparing multiple groups, or two-tailed Student's T test was performed when comparing 2 groups using GraphPad Prism 4 software (GraphPad Software, San Diego, CA). *, ** and *** indicate P values below 0.05, 0.01 and 0.001 when using Bonferroni's Multiple comparison test, and ### indicate P values below 0.001 when using two-tailed Student's T test. Data are represented as mean ± 1 standard deviation unless otherwise indicated.

## Supporting Information

S1 Fig Additional cell viability and delivery efficiency data for primary murine immune cells. **A**—Representative figures of uptake of 3 kDa and 70 kDa dextran and antibody to murine primary immune cells. The gating used to calculate delivery efficiency values is shown. These data correspond to experiments presented in **Fig 2 (main text).** Grey histograms represent untreated cells, black represents cells that were exposed to the materials but not treated by the device, red represents cells that were treated by the device in the presence of the target biomolecules. **Gating Strategy:** To quantify delivery efficiency of a particular fluorophore, a gate is created on the corresponding channel such that an endocytosis control case has a 5–10% ‘delivery efficiency’. This strategy relies on the endocytosis control to account for any surface binding/endocytosis effects of the fluorophores and it is assumed that any observed increase of fluorescence beyond the set threshold is due to intracellular delivery by the CellSqueeze device. 5–10% was chosen instead of a lower threshold in order to ensure that we do not undercount the delivery efficiency contribution from cells that received enough dye to shift relative to the original distribution but not enough to cross a more conservative gate threshold. **B**—Cell viability data corresponding to the experiments presented in **Fig 2**. *** indicated p < 0.001 when comparing viability of cells treated with 30–4 device to no device or untreated cases. Changes in viability of B cells and myeloid cells treated with the device were not significantly different from the untreated or no device cases. **C—**Delivery of dextran and antibodies to bone marrow-derived dendritic cells (BMDCs). BMDCs were generated from C57BL6 mice by culturing bone marrow cells in GM-CSF containing media for 8 days. Cascade blue-labeled 3 kDa dextran, fluorescein-labeled 70 kDa dextran, and APC-labeled IgG1 were delivered using two device designs, 10–6 and 30–6. **D**—Correlation of antibody and dextran delivery. Dextran (3 kDa and 70 kDa) and antibody delivery to T cells using the 30–4 device (**red dots**) compared to incubation with the material, i.e. no device (**black dots**).
(TIF)

S2 Fig Additional cell viability, delivery and knockdown data for primary human immune cells. **A—**Delivery (***left***), representative flow cytometry histograms from a 30–4 device (***middle***) and viability of human CD4+ T cells (***right***) used to deliver dextrans and antibodies to human CD4^+^ T cells. Cascade blue-labeled 3 kDa dextran, fluorescein-labeled 70kDa dextran, and APC-labeled IgG1 were delivered using 2 device designs or by Amaxa nucleofection. Cells that pass through the device have reduced viability when compared to untreated controls, but do better than cells that have undergone nucleofection. One-way ANOVA followed by Boneferroni's test was used to calculate statistical significance. * indicates p < 0.05 and *** indicates p < 0.001. Other groups of comparison did not show significantly different viability (i.e. 10–4 compared to untreated or 30–4, and 30–4 compared to nucleofection). Note that the antibody ‘delivery’ shown by nucleofection could potentially be an artifact of protein damage. Follow-up experiments wherein the antibody is exposed to the nucleofection treatment in the absence of cells, and subsequently mixed with untreated cells, yielded mixed results with some data indicating that antibody damage due to the fields alone could be sufficient to yield a false-positive. Moreover the 3kDa and 70kDa dextran, both smaller molecules than the antibody, were not delivered as effectively. There is also limited published evidence that electroporation is effective for protein delivery (18,19). Note: 30-5x5, 10-4x2, 10-5-4-5, 10-6-4-6, 30-5-4-5, and 10-4x5 designs were also tested for murine and human T cells, but none was superior to the performance of 30–4 (data not shown). **B—**Delivery (***top***) and viability (***bottom***) for human MDDCs. Cascade blue labeled 3kDa dextran, fluorescein labeled 70kDa dextran, and APC labeled IgG1 isotype control antibodies were delivered using 6 different device designs and using Amaxa nucleofection. Viability and delivery results were measured immediately after treatment. **C**—siRNA delivery (***top***) and protein knockdown (***bottom***) in human T cells. Alexa 488 or Alexa 647 labeled siRNA and 3kDa cascade blue labeled dextran were delivered simultaneously to human CD4 T cells by a 10-4i device and murine B cells by a 30-5x5i device. The data indicate that delivery of the two materials correlates closely. This result is consistent with the proposed diffusive delivery mechanism, i.e. delivery efficacy is mostly dependent on material size rather than chemical structure. For knockdown experiments (***bottom***), siRNA against CD45RA was delivered to human T cells by a 10–4 device. Knockdown was measured by flow cytometry 72 hours post-treatment. **D**—mRNA knockdown (***left***) data corresponding to **Fig 2B** as measured by PCR 48 hours after delivery. Expression levels of CD4 in CD4^+^ human T cells over 2 weeks post-treatment (***middle***) as measured by flow cytometry. CD3 levels were also measured as a control gene (***right***).
(TIF)

S3 Fig Delivery to primary human monocytes, B cells and DCs. **A—**Delivery of dextran to human monocytes. Monocytes were derived from human blood. Cascade blue labeled 3kDa dextran, and fluorescein labeled 70kDa dextran were delivered using four different device designs at two different operating pressures. The 0psi case corresponds to controls that were only exposed to dextran but not treated by the device. Viability was measured by propidium iodide staining. **B**—Delivery of dextran to human B cells. B cells were derived from human blood. Cascade blue labeled 3kDa dextran, and fluorescein labeled 2MDa dextran were delivered using five different device designs at two different operating pressures. The 0psi case corresponds to controls that were only exposed to dextran but not treated by the device. Viability was measured by propidium iodide staining. **C**—Protein levels of DC-Sign 72hrs after treatment. Protein knockdown was measured across 6 different device designs and compared to nucleofection. Note that nucleofection appears to cause ~50% non-specific knockdown of DC-Sign even in the case of control siRNA delivery. This could indicate potential off-target effects due to the electroporation treatment.
(TIF)

S1 Table Device designs tested and operating parameters. **A.** Library of tested device designs. Note that not all designs were tested for all cell types. The first number indicates constriction length, subsequent numbers preceded by a dash indicate the width of a constriction. If there are multiple identical constrictions in series it is indicated by an ‘x’ followed by the number of constrictions. For example, 10-5-4-5 contains 3 10μm long constrictions in series with widths of 5 μm, 4 μm, and 5 μm. 10-4x5 contains 5 10 μm long constrictions in series, each with a 4 μm width. Note: The multiple constriction designs were used to explore if there were any advantages to squeezing a cell multiple times within the same delivery cycle using the same or different sized constrictions. Although some differences in performance were observed, i.e. multiple constrictions of the same dimension yielded higher delivery and lower viability relative to a single constriction, none of the tested multi-constriction chips emerged as a more effective alternative to a single constriction chip. This parameter may warrant further investigation in future studies to deepen our understanding of its relevance to the delivery process and its potential to optimize delivery in certain cell types. **B.** This table summarizes the results from the tested device designs. A ‘*’ indicates that the device design was able to achieve >20% delivery AND >30% viability with the listed cell type. ‘LD’ indicates ‘low delivery’ which means the delivery efficiency with these chip types was below the desired threshold. ‘LV’ indicates ‘ low viability’ which means the viability was below the desired threshold. **C.** Delivery parameters and their influence on performance
(DOCX)
