# Supplementary figures and images for: Ex Vivo Cytosolic Delivery of Functional Macromolecules to Immune Cells
Source: PLoS One. 2015 Apr 13;10(4):e0118803. doi: 10.1371/journal.pone.0118803 (PMC4395260; doi:10.1371/journal.pone.0118803)

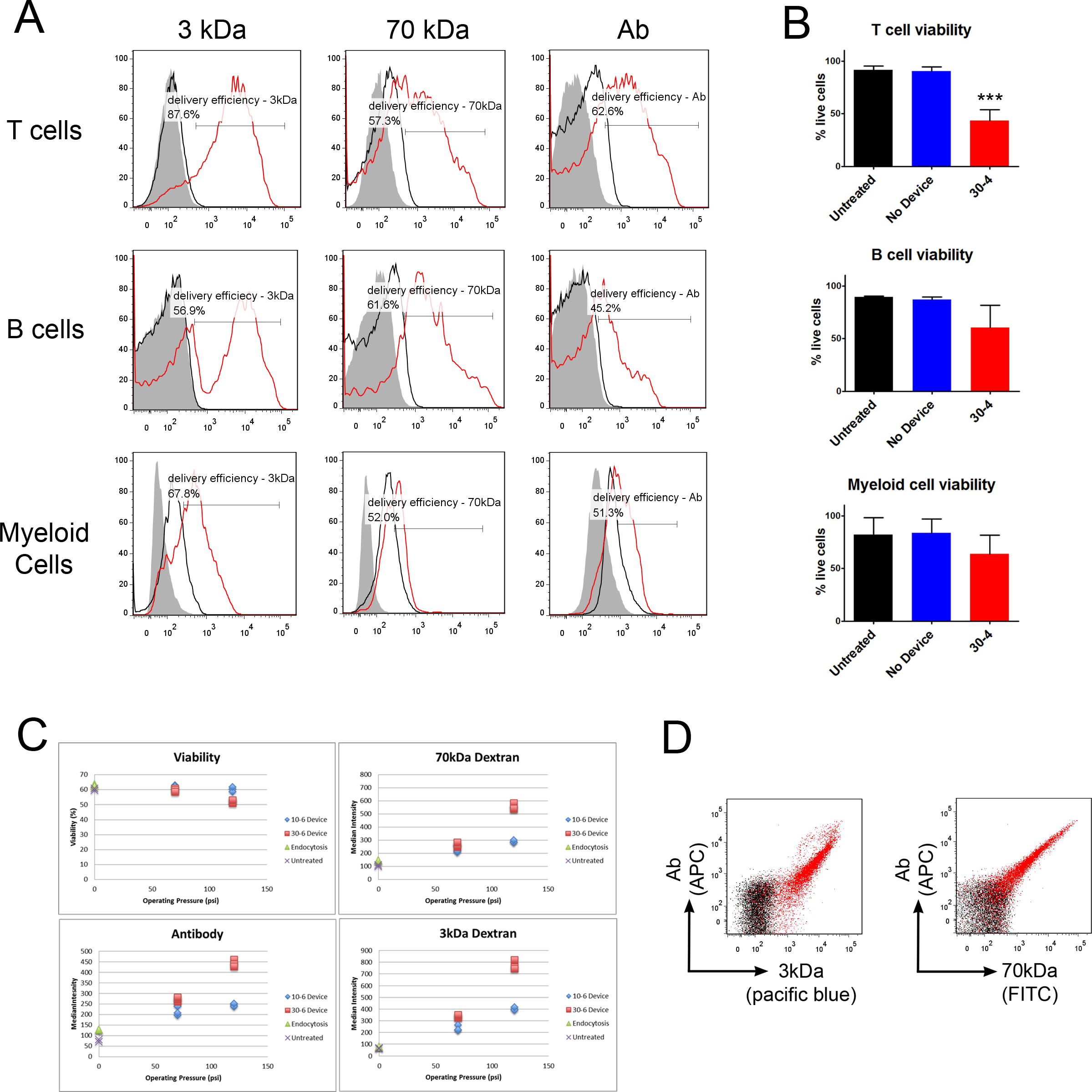

Supplement: S1 Fig — A—Representative figures of uptake of 3 kDa and 70 kDa dextran and antibody to murine primary immune cells. The gating used to calculate delivery efficiency values is shown. These data correspond to experiments presented in Fig 2 (main text). Grey histograms represent untreated cells, black represents cells that were exposed to the materials but not treated by the device, red represents cells that were treated by the device in the presence of the target biomolecules. Gating Strategy: To quantify delivery efficiency of a particular fluorophore, a gate is created on the corresponding channel such that an endocytosis control case has a 5–10% ‘delivery efficiency’. This strategy relies on the endocytosis control to account for any surface binding/endocytosis effects of the fluorophores and it is assumed that any observed increase of fluorescence beyond the set threshold is due to intracellular delivery by the CellSqueeze device. 5–10% was chosen instead of a lower threshold in order to ensure that we do not undercount the delivery efficiency contribution from cells that received enough dye to shift relative to the original distribution but not enough to cross a more conservative gate threshold. B—Cell viability data corresponding to the experiments presented in Fig 2. *** indicated p < 0.001 when comparing viability of cells treated with 30–4 device to no device or untreated cases. Changes in viability of B cells and myeloid cells treated with the device were not significantly different from the untreated or no device cases. C—Delivery of dextran and antibodies to bone marrow-derived dendritic cells (BMDCs). BMDCs were generated from C57BL6 mice by culturing bone marrow cells in GM-CSF containing media for 8 days. Cascade blue-labeled 3 kDa dextran, fluorescein-labeled 70 kDa dextran, and APC-labeled IgG1 were delivered using two device designs, 10–6 and 30–6. D—Correlation of antibody and dextran delivery. Dextran (3 kDa and 70 kDa) and antibody delivery to T cells usi [file pone.0118803.s001.tif]

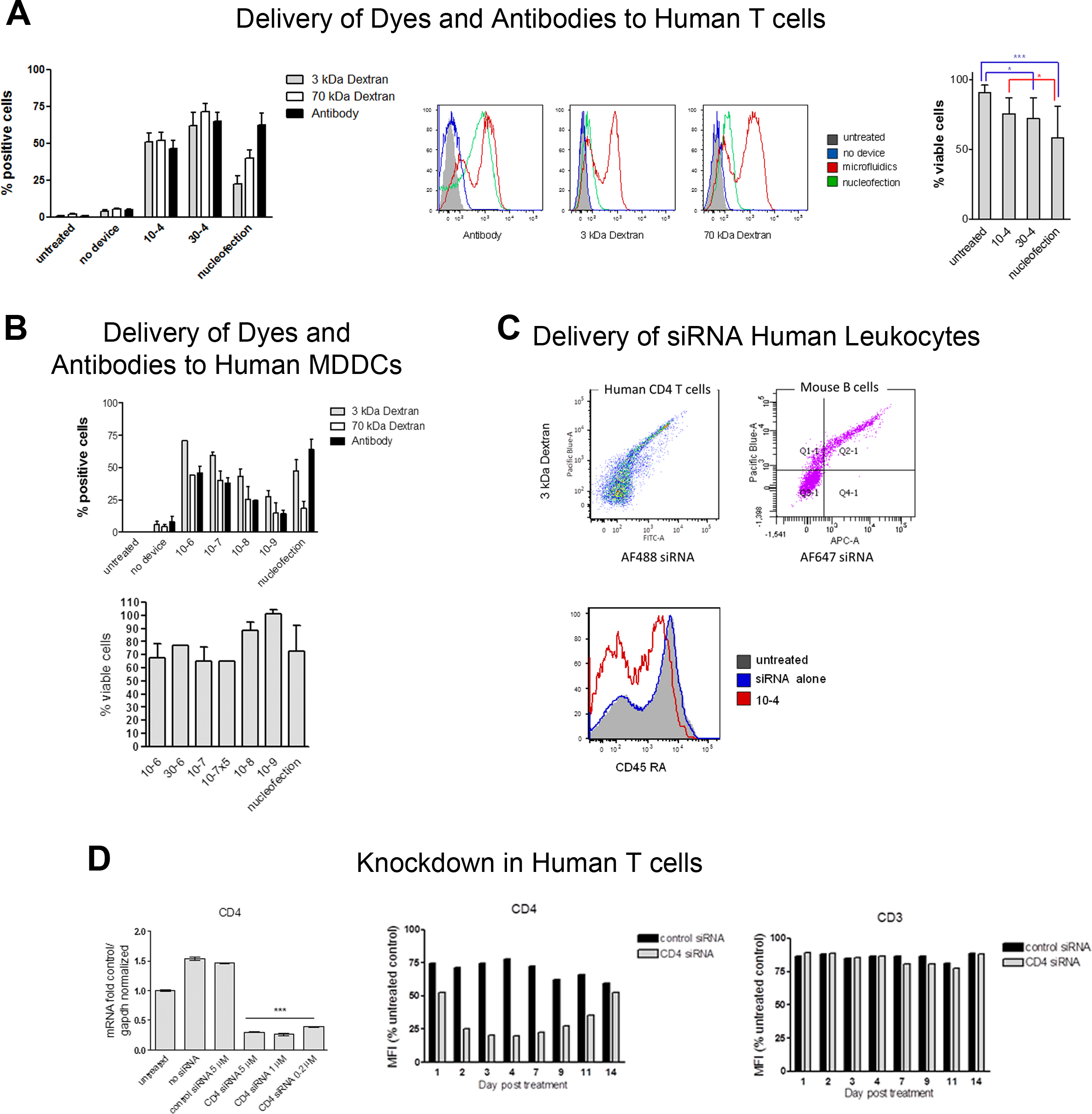

Supplement: S2 Fig — A—Delivery (left), representative flow cytometry histograms from a 30–4 device (middle) and viability of human CD4+ T cells (right) used to deliver dextrans and antibodies to human CD4+ T cells. Cascade blue-labeled 3 kDa dextran, fluorescein-labeled 70kDa dextran, and APC-labeled IgG1 were delivered using 2 device designs or by Amaxa nucleofection. Cells that pass through the device have reduced viability when compared to untreated controls, but do better than cells that have undergone nucleofection. One-way ANOVA followed by Boneferroni's test was used to calculate statistical significance. * indicates p < 0.05 and *** indicates p < 0.001. Other groups of comparison did not show significantly different viability (i.e. 10–4 compared to untreated or 30–4, and 30–4 compared to nucleofection). Note that the antibody ‘delivery’ shown by nucleofection could potentially be an artifact of protein damage. Follow-up experiments wherein the antibody is exposed to the nucleofection treatment in the absence of cells, and subsequently mixed with untreated cells, yielded mixed results with some data indicating that antibody damage due to the fields alone could be sufficient to yield a false-positive. Moreover the 3kDa and 70kDa dextran, both smaller molecules than the antibody, were not delivered as effectively. There is also limited published evidence that electroporation is effective for protein delivery (18,19). Note: 30-5x5, 10-4x2, 10-5-4-5, 10-6-4-6, 30-5-4-5, and 10-4x5 designs were also tested for murine and human T cells, but none was superior to the performance of 30–4 (data not shown). B—Delivery (top) and viability (bottom) for human MDDCs. Cascade blue labeled 3kDa dextran, fluorescein labeled 70kDa dextran, and APC labeled IgG1 isotype control antibodies were delivered using 6 different device designs and using Amaxa nucleofection. Viability and delivery results were measured immediately after treatment. C—siRNA delivery (top) and protein knockdown (bottom) in huma [file pone.0118803.s002.tif]

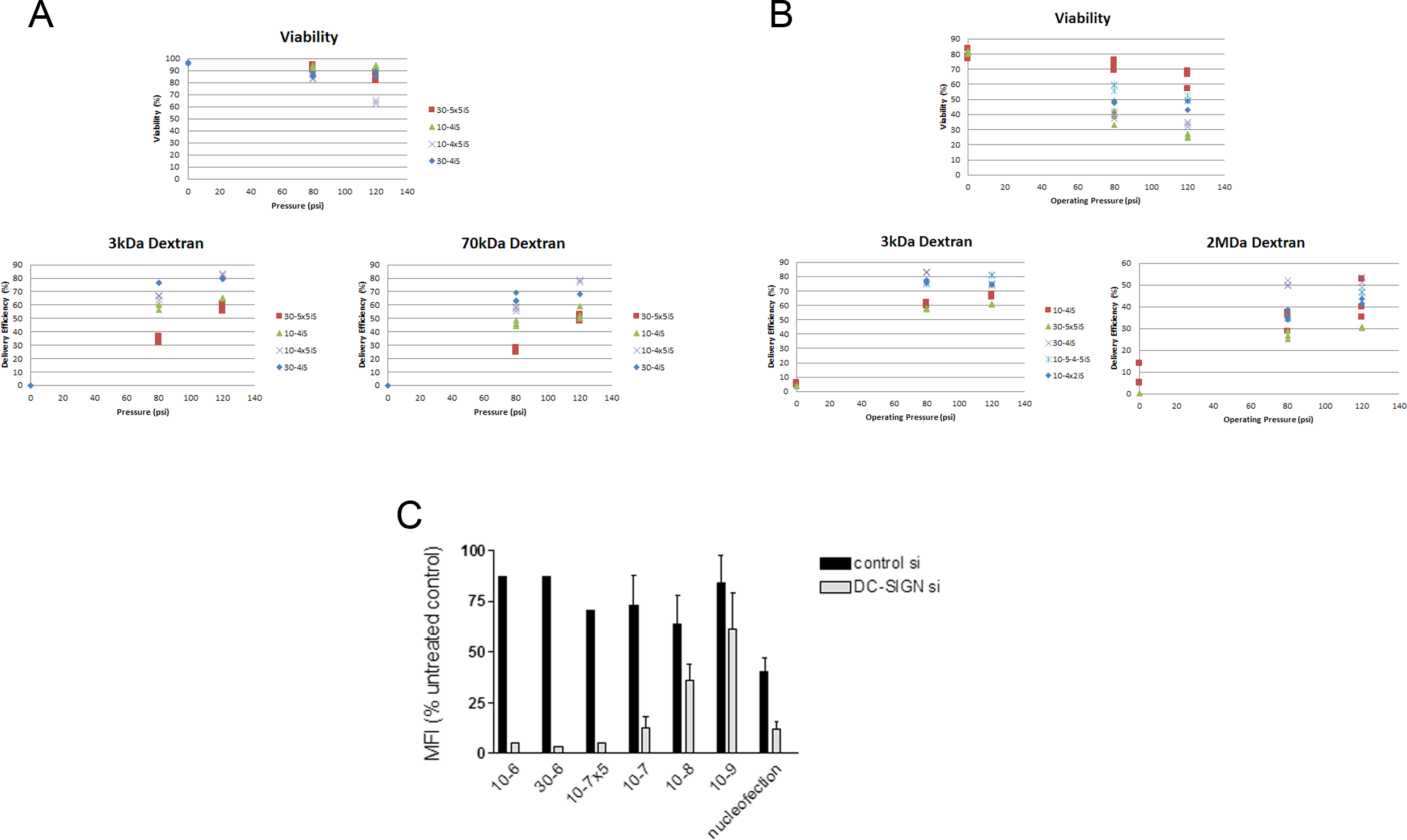

Supplement: S3 Fig — A—Delivery of dextran to human monocytes. Monocytes were derived from human blood. Cascade blue labeled 3kDa dextran, and fluorescein labeled 70kDa dextran were delivered using four different device designs at two different operating pressures. The 0psi case corresponds to controls that were only exposed to dextran but not treated by the device. Viability was measured by propidium iodide staining. B—Delivery of dextran to human B cells. B cells were derived from human blood. Cascade blue labeled 3kDa dextran, and fluorescein labeled 2MDa dextran were delivered using five different device designs at two different operating pressures. The 0psi case corresponds to controls that were only exposed to dextran but not treated by the device. Viability was measured by propidium iodide staining. C—Protein levels of DC-Sign 72hrs after treatment. Protein knockdown was measured across 6 different device designs and compared to nucleofection. Note that nucleofection appears to cause ~50% non-specific knockdown of DC-Sign even in the case of control siRNA delivery. This could indicate potential off-target effects due to the electroporation treatment. (TIF) [file pone.0118803.s003.tif]
