# Supplementary material for: Ex Vivo Cytosolic Delivery of Functional Macromolecules to Immune Cells
Source: PLoS One. 2015 Apr 13;10(4):e0118803. doi: 10.1371/journal.pone.0118803 (PMC4395260; doi:10.1371/journal.pone.0118803)
Supplement: S1 Table — A. Library of tested device designs. Note that not all designs were tested for all cell types. The first number indicates constriction length, subsequent numbers preceded by a dash indicate the width of a constriction. If there are multiple identical constrictions in series it is indicated by an ‘x’ followed by the number of constrictions. For example, 10-5-4-5 contains 3 10μm long constrictions in series with widths of 5 μm, 4 μm, and 5 μm. 10-4x5 contains 5 10 μm long constrictions in series, each with a 4 μm width. Note: The multiple constriction designs were used to explore if there were any advantages to squeezing a cell multiple times within the same delivery cycle using the same or different sized constrictions. Although some differences in performance were observed, i.e. multiple constrictions of the same dimension yielded higher delivery and lower viability relative to a single constriction, none of the tested multi-constriction chips emerged as a more effective alternative to a single constriction chip. This parameter may warrant further investigation in future studies to deepen our understanding of its relevance to the delivery process and its potential to optimize delivery in certain cell types. B. This table summarizes the results from the tested device designs. A ‘*’ indicates that the device design was able to achieve >20% delivery AND >30% viability with the listed cell type. ‘LD’ indicates ‘low delivery’ which means the delivery efficiency with these chip types was below the desired threshold. ‘LV’ indicates ‘ low viability’ which means the viability was below the desired threshold. C. Delivery parameters and their influence on performance (DOCX) [file pone.0118803.s004.docx]

**A.**

| **Tested Library of constriction designs** | | | |
| --- | --- | --- | --- |
| 10-4 | 10-6-4-6 | 10-6 | 10-7x5 |
| 10-4x2 | 30-4 | 30-6 | 10-7 |
| 10-4x5 | 30-5-4-5 | 50-6 | 10-8 |
| 10-5-4-5 | 30-5x5 | 10-6x5 | 10-9 |

**B.**

**C.**

| Constriction Design | Constriction geometry (specifically length and width) and number of constrictions in series affect delivery efficiency and cell viability. Longer, narrower, and more numerous constrictions typically result in more effective delivery but can lead to lower viability |
| --- | --- |
| Operating Pressure | The operating pressure of the system determines the speed at which cells move through the channels and are deformed. Higher speeds lead to more rapid deformation, which can result in higher delivery efficiency and potentially lower viability. |
| Flow Buffer | The buffer in which cells are suspended during treatment can affect cell health and may potentially interact with the biomolecule being delivered (e.g. serum proteins may bind certain materials). One known effect on delivery is mediated by calcium. The presence of calcium ions in the running buffer speeds up membrane repair post-treatment. Operating in calcium-free buffers can increase delivery at the risk of reducing viability. |
| Operating Temperature | Lower temperatures (i.e. on ice) improve delivery efficiency. Temperature could influence many parameters. One possibility is that low temperatures retard membrane repair post-treatment. |
